# SEDENTARY BEHAVIORS AND COGNITION: DOES THE TYPE OF BEHAVIOR MATTER?

**DOI:** 10.1093/geroni/igad104.3114

**Published:** 2023-12-21

**Authors:** Mariana Wingood, Erin Bouldin, Nancy Gell

**Affiliations:** Wake Forest University, Winston-Salem, North Carolina, United States; University of Utah, Salt Lake City, Utah, United States; University of Vermont, Burlington, Vermont, United States

## Abstract

Older adults spend 80% of their waking hours performing sedentary behaviors (SBs) that are either mentally active (e.g., using computers, socializing, and completing hobbies) or passive (e.g., television viewing and resting). Cross-sectional studies report an association between passive SB participation and cognitive impairments. However, longitudinal impact of baseline SB participation on cognition is unclear. We addressed this gap by using the National Health and Aging Trends Study (NHATS) dataset to run competing risks proportional hazards models. In our models, participants were identified as having an event (i.e., outcome) if their cognition scores dropped ≤ 1.5 standard deviations (SD) below the mean during follow-up (rounds 7-11, years 2017-2021). Competing risks included moving to long-term care facilities or dying. Our analyses included 1,574 community-dwelling older adults (mean age 84, SD = 7.1) who completed the sedentary module included in round 6 (year 2016) and had intact orientation, recall, and executive function at baseline. The majority of participants were female (55%) and non-Hispanic white (78%), and reported good or excellent health (80%). We identified that participating in active SBs had a protective effect on orientation, recall, and executive function (subdistribution hazard ratios [SHR]= 0.28-0.46, p< 0.001-0.02). Activities that had significant protective effects across all models included using computers (SHR =0.15-0.46, p=< 0.001-0.001) and socializing (SHR=0.27-0.52, p= < 0.001-0.05). Passive SBs did not have significant SHRs. Our identified protective effects highlight the potential of preventing cognitive impairments through active SB programs. Intervention studies are needed to develop program protocols and identify effectiveness of active SB programs.

